# Genome editing approaches using reproductive cells/tissues in flowering plants

**DOI:** 10.3389/fgeed.2022.1085023

**Published:** 2023-01-11

**Authors:** Erika Toda, Norio Kato, Tetsuya Higashiyama, Takashi Okamoto

**Affiliations:** ^1^ Department of Biological Sciences, The University of Tokyo, Tokyo, Japan; ^2^ Department of Biological Sciences, Tokyo Metropolitan University, Tokyo, Japan

**Keywords:** CRISPR/Cas9, embryo, initiating cell, targeted mutagenesis, plant, pollen grain, shoot apical meristem, zygote

## Abstract

Targeted mutagenesis *via* programmable nucleases including the clustered regulatory interspaced short palindromic repeats (CRISPR)/CRISPR-associated protein 9 (Cas9) (CRISPR/Cas9) system has been broadly utilized to generate genome-edited organisms including flowering plants. To date, specific expression of Cas9 protein and guide RNA (gRNA) in reproductive cells or tissues is considered one of the most effective genome-editing approaches for heritable targeted mutagenesis. In this report, we review recent advances in genome editing methods for reproductive cells or tissues, which have roles in transmitting genetic material to the next-generation, such as egg cells, pollen grains, zygotes, immature zygotic embryos, and shoot apical meristems (SAMs). Specific expression of Cas9 proteins in initiating cells efficiently induces targeted mutagenesis *via* *Agrobacterium*-mediated *in planta* transformation. In addition, genome editing by direct delivery of CRISPR/Cas9 components into pollen grains, zygotes, cells of embryos and SAMs has been successfully established to generate genome-edited plant lines. Notably, DNA-free genome editing by the delivery of Cas9-gRNA ribonucleoproteins (RNPs) is not associated with any legislative concerns about genetically modified organisms. In summary, the genome editing methods for reproductive cells or tissues have enormous potential for not only basic studies for plant reproduction but also applied sciences toward molecular plant breeding.

## Introduction

Technology involving targeted mutagenesis using programmable nucleases, such as zinc-finger nucleases (ZFNs) (Urnov et al., 2010), transcription activator-like effector nucleases (TALENs) (Cermak et al., 2011), and RNA-guided endonucleases (RGENs), has been rapidly developing and has enormous potential to accelerate basic and applied sciences. The programmable nucleases produce double-strand breaks (DSBs) at target sites in genomic DNA, and these DSBs can be repaired by two independent pathways: non-homologous end-joining (NHEJ) and homology-directed repair (HDR) (Roth and Wilson, 1986; Puchta et al., 1993; Moore and Haber, 1996; Jasin and Rothstein, 2013).

In RGENs, the clustered regulatory interspaced short palindromic repeats (CRISPR)/CRISPR-associated protein 9 (Cas9) (CRISPR/Cas9) system has paved the way for the development of rapid and cost-effective procedures to create new mutant populations in plants (Belhaj et al., 2013; Voytas 2013). In general, the CRISPR/Cas9 expression cassette and selectable marker are integrated into plasmid DNA, and the constructs are delivered into plant cells *via Agrobacterium tumefaciens*-mediated transformation or particle bombardment (Kumar and Jain, 2015; Luo et al., 2016). Plant lines that have integrated the constructs into genomic DNA are selected by the selectable marker and genome-edited plant lines can be screened by the sequencing of target sites. However, constitutive expression of CRISPR/Cas9 in the plant life cycle generates a large proportion of non-heritable mutations in somatic cells (Feng et al., 2014), and increases the likelihood of DNA cleavage at non-specific loci, so-called off-target modifications, in plant genome editing (Lawrenson et al., 2015). To induce heritable mutations and reduce off-target modifications, a genome editing system through CRISPR/Cas9 expression under a reproductive cell- or tissue-specific promoter has been developed, which we will summarize later in this review.

In animals, to produce genetically heritable traits of interest, *in vitro* transcribed RNAs encoding Cas9 and gRNA are directly delivered into eggs or zygotes, resulting in the highly efficient production of genetically modified animals (Hwang et al., 2013; Wang et al., 2013). In angiosperms, although female gamete, zygote, and embryo exist in the embryo sac deeply embedded in ovular tissue (Russell 1992; Raghavan 2003), such reproductive cells/tissues isolated from flowers have been successfully used as targets for the direct delivery of CRISPR/Cas9 vectors or preassembled Cas9 protein-guide RNA (gRNA) ribonucleoproteins (RNPs). Moreover, shoot apical meristems (SAMs) including a subepidermal cell layer, L2, from which germ cells later develop during floral organogenesis have also been target tissues for an inheritable genome editing approach. In this mini review, we summarize the current approaches of genome editing using plant reproductive cells/tissues, such as egg cell, pollen grain, zygote, embryo, and SAM, based on the frequencies of targeted mutagenesis and off-target mutations.

### Cell/tissue-specific Cas9 expression in *Arabidopsis* initiating cells

**FIGURE 1:**
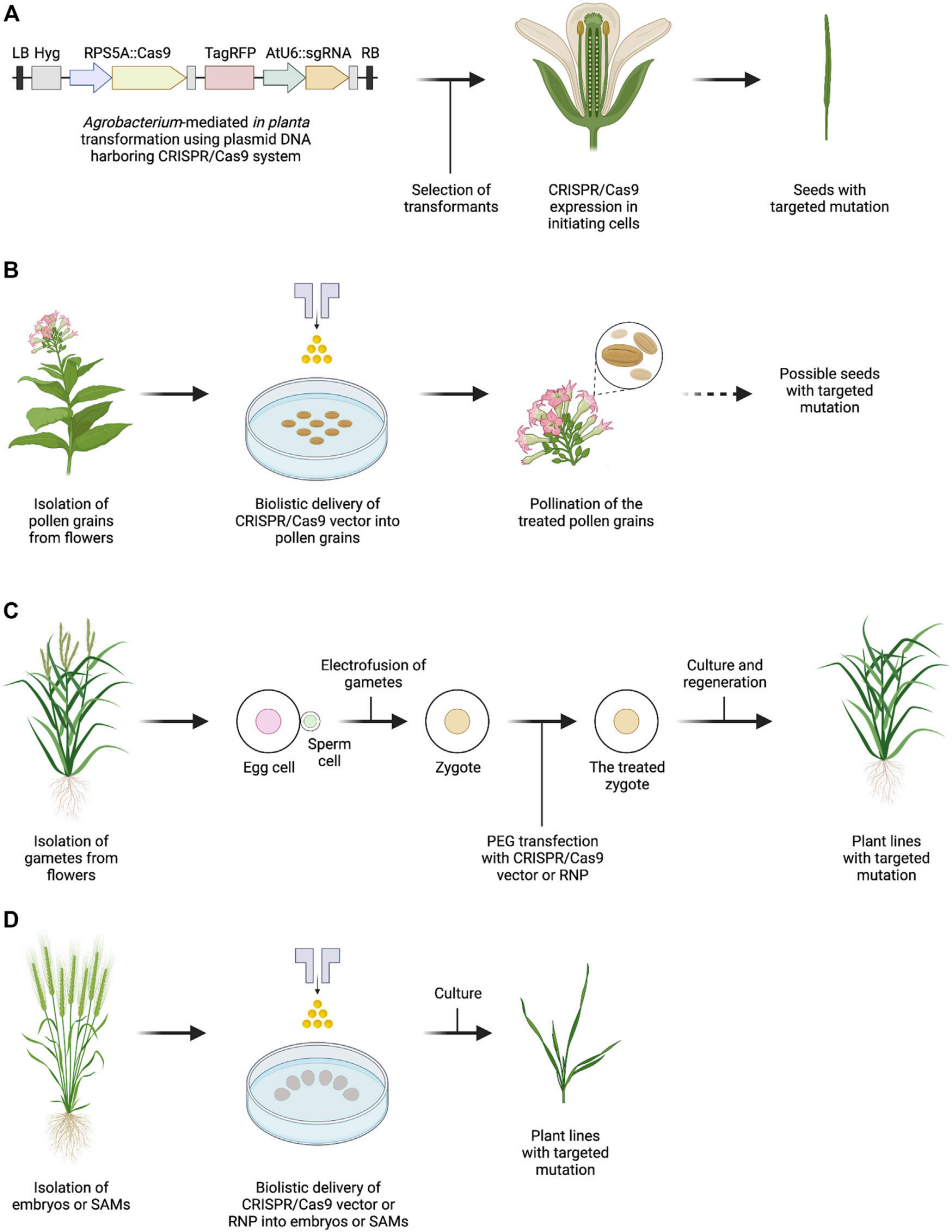
Outline of genome editing methods for plant reproductive cells or tissues. **(A)** *Agrobacterium*-mediated in planta transformation with CRISPR/Cas9 vector in which promoters for Cas9 expression are specifically expressed in *Arabidopsis* initiating cells. Vector information is taken with reference to Tsutsui and Higashiyama (2017). **(B)** Biolistic delivery of CRISPR/Cas9 vector into tobacco pollen grains. The dotted arrow indicates a procedure that has not been experimentally tested. **(C)** PEG-Ca^2+^-mediated transfection of rice zygotes with CRISPR/Cas9 vector or Cas9-gRNA RNP. **(D)** Biolistic delivery of CRISPR/Cas9 vector or Cas9-gRNA RNP into cells of wheat embryos and SAMs. The figure was created with BioRender.com.

In general, *Agrobacterium*-mediated *in planta* transformation has been applied to introduce the CRISPR/Cas9 expression cassette into *Arabidopsis*. Ubiquitously expressed Cas9 protein and gRNA generate targeted gene modifications with high efficiency; however, only the gene modification generated in reproductive cells can be transmitted to the next-generation (Feng et al., 2014). To efficiently induce inheritable targeted mutations, specific promoters for the germline (*Elongation Factor-1α*(*EF1α*) promoter; Osakabe et al., 2016) and egg cell (*EC* promoter; Wang et al., 2015; Zheng et al., 2020, and *DD45* promoter; Mao et al., 2016) have been successfully used for the exogenous expression of Cas9-gRNA complexes in reproductive cells of *Arabidopsis*. Moreover, the *RIBOSOMAL PROTEIN S5A* (*RPS5A*) promoter, which is constitutively active at the beginning of the process of egg cell formation, was shown to be efficient for driving the expression of Cas9 in *Arabidopsis* female germ cells (Figure 1A; Tsutsui and Higashiyama, 2017). In addition to preferential Cas9 expression in female gametes, the *SPOROCYTELESS* (*SPL*) genomic expression cassette, which is specifically expressed in sporogenous cells and microsporocytes, has been used for germline-specific Cas9 expression in male *Arabidopsis* gametocytes (Mao et al., 2016). Furthermore, the *YAO* promoter, which is preferentially active in the embryo sac, embryo, endosperm, pollen and SAM, has been used for the expression of Cas9 (Yan et al., 2015). These approaches efficiently and preferentially generate progeny with a high diversity of mutations at the targeted locus.

### *In planta* gene targeting using egg cell-specific Cas9 expression in *Arabidopsis*

In addition to NHEJ-based genome editing, a cell/tissue-specific promoter for initiating cells has been applied for Cas9 expression to induce heritable gene targeting (GT) in *Arabidopsis*. In this strategy, parental lines expressing Cas9 under the egg cell- and early embryo-specific *DD45* promoter were used in combination with the delivery of HDR donor DNA to increase genome editing activity, resulting in high efficiency of GT of ca. 5.3%–9.1% (Miki et al., 2018). Wolter et al. (2018) also demonstrated that the use of Cas9 under a ubiquitin promoter leads to seeds harboring GT events with a low frequency, whereas the use of Cas9 controlled under an egg cell-specific promoter was the most efficient approach, achieving a frequency of around 1% of the seeds. These results indicate that the use of a reproductive cell- or tissue-specific promoter is an effective genome editing approach to achieve heritable targeted mutagenesis *via* either NHEJ or HDR by *Agrobacterium*-mediated *in planta* transformation.

### Direct delivery of macromolecules into pollen grains

Particle bombardment can be used to deliver macromolecules into various tissues such as immature zygotic embryos, leaf disks, and calli, and is not limited by plant-host range (Altpeter et al., 2005). Pollen grains are structurally simple tissue containing male germ cells, and are easily isolated from anthers. Therefore, the direct delivery of CRISPR/Cas9 vector into *Nicotiana benthamiana* pollens *via* biolistic delivery triggers genome editing of the pollen grains, and the bombarded pollen enables the elongation of pollen tubes and delivery of sperm cells into the embryo sac (Table 1; Nagahara et al., 2021). Although biolistic delivery conditions and seed detection methods should be optimized, this delivery approach using pollen grains may be broadly applicable to obtaining progeny with targeted mutations (Figure 1B). Furthermore, procedures for delivering exogenous materials into pollen grains have been demonstrated with various approaches (Eapen et al., 2011; Zhao et al., 2017; Bhowmik et al., 2018). Recently, Lei et al. (2021) has reported genome editing using pollen-specific Cas9 expression *via Agrobacterium* vacuum infiltration in cotton. In addition, magnetic nanoparticles have been reported as a novel physiological procedure for transforming pollen grains (Zhao et al., 2017), although pollen magnetofection can only be applied in cotton pollen (Vejlupkova et al., 2020).

**TABLE 1:** Genome editing by direct delivery of CRISPR/Cas9 components into plant reproductive cells or tissues.

Cells or tissues used for genome editing	Plant species	CRISPR/Cas9 component	Methods for CRISPR/Cas9 delivery	Target genes	Off-target detection (Target gene)	Efficiency of targeted mutagenesis	References
Pollen	*N. benthamiana*	DNA	Particle bombardment	*PDS3*	—	—	Nagahara et al. (2021)
Zygote	*Oryza sativa*	DNA	PEG-Ca^2+^ transfection	*DL, PRR37*	—	4.0%–25.0%	Toda et al. (2019)
		RNP		*DL, GW7, GCS1*		13.6%–64.3%	
Embryo	*Zea mays*	DNA	Particle bombardment	*LIG1, MS26, MS45, ALS2*	2.0% (*MS45*)	4.0%	Svitashev et al. (2016)
		RNP		*LIG1, MS26, MS45, ALS2*	0% (*MS45*)	2.4%–9.7%	
	*Triticum aestivum*	DNA	Particle bombardment	*TaGW2*	3.8% (*TaGW2-A1*)	4.1%–4.4%	Liang et al. (2017)
		RNP			n.d. (*TaGW2-A1*)	2.2%–4.4%	
SAM	*Triticum aestivum*	DNA	Particle bombardment	*TaGASR7*	—	5.2%	Hamada et al. (2018)
		RNP		*SD1, TaOr, TaQsd1, TaHRGPL1*	n.d. (*SD1*)	1.9%–8.3%	Kumagai et al. (2022)

*n.d., not detected.

### PEG-Ca^2+^-mediated transfection of zygotes with CRISPR/Cas9 components

In animals, to produce genetically heritable traits of interest, *in vitro* transcribed Cas9 mRNA and sgRNA or preassembled Cas9 protein-sgRNA complexes are delivered into zygotes by direct injection, resulting in the production of bi-allelic mutants with high efficiency (Gratz et al., 2013; Hwang et al., 2013; Wang et al., 2013). In angiosperms, a genome editing system *via* direct delivery of CRISPR/Cas9 vectors or Cas9-gRNA RNPs into rice zygotes has recently been developed (Figure 1C; Toda et al., 2019). CRISPR/Cas9 vectors or Cas9-gRNA RNPs were transfected into rice zygotes produced by *in vitro* fertilization (IVF) of isolated gametes *via* polyethylene glycol-calcium (PEG-Ca^2+^)-mediated transfection (Koiso et al., 2017). Thereafter, the treated zygotes were cultured in the absence of selection agents, resulting in the regeneration of rice plants with targeted mutations, at frequencies in the range of ca. 4%–64% (Table 1; Toda et al., 2019). In addition to rice, IVF systems have been established in maize (Kranz and Lörz, 1993) and wheat (Maryenti et al., 2019), suggesting that a zygote-based genome editing approach would be applicable to other crop species.

### Biolistic delivery of CRISPR/Cas9 components into cells of embryos and SAMs

Although particle bombardment delivery of CRISPR/Cas9 expression cassette into immature zygotic embryos has showed successful genome editing, Mendelian segregation distortion was observed in progeny plants (Svitashev et al., 2015). One possibility is that constitutive expression of CRISPR/Cas9 lead to somatic mutations, resulting in chimeric plants (Feng et al., 2014; Svitashev et al., 2015). Therefore, to overcome the issue, genome editing approach by direct delivery of Cas9-gRNA RNPs into cells of embryos has been developed in maize, and the frequencies of targeted mutagenesis were in the range of 2.4%–9.7% (Table 1; Svitashev et al., 2016). Similarly, 2.2%–4.4% of regenerated plants contained target mutations were obtained in wheat (Figure 1D; Table 1; Liang et al., 2017).

In addition to embryos, *in planta* transformation using biolistic delivery of CRISPR/Cas9 vector to wheat SAMs, which maintain the potential to develop into flower organs, has been reported as an *in planta* particle bombardment (iPB) method, with targeted mutations in 5.2% of the bombarded plants (Figure 1D; Table 1; Hamada et al., 2018). Notably, the iPB method is a non-culture method that does not require callus culture and regeneration procedures. Furthermore, a system of directly delivering Cas9-gRNA RNPs into wheat SAMs has recently been established, and no mutations were found at the potential off-target sites (Table 1; Kumagai et al., 2022).

## Discussion

In animals, genome editing approaches have been established using germline cells, zygotes, and embryos to obtain genome-edited organisms by inducing heritable genetic changes (Cooper et al., 2017; Lea and Niakan 2019; Koslova et al., 2020). In this mini review, we described genome editing approaches using plant reproductive cells or tissues toward efficient and precise genome editing. In *Arabidopsis*, specific and sufficient expression of Cas9 proteins in initiating cells, such as germ cells, egg cells, and SAMs, is crucial for efficient targeted mutagenesis through *Agrobacterium*-mediated *in planta* transformation (reviewed in Osakabe and Osakabe, 2017).

In addition to *Agrobacterium*-mediated methods, genome editing *via* direct delivery of CRISPR/Cas9 components into plant cells or tissues has been developed. Notably, DNA-free genome editing, which can avoid the introduction of foreign DNA sequences into genomic DNA, has been achieved by the direct delivery of Cas9-gRNA RNP into somatic protoplasts *via* PEG-Ca^2+^-mediated transfection, such as in tobacco, *Arabidopsis*, lettuce, rice (Woo et al., 2015), *Petunia* (Subburaj et al., 2016), grapevine, apple (Malnoy et al., 2016), potato (Andersson et al., 2018), and tomato (Liu et al., 2022). Although a somatic protoplast-based genome editing can use abundant isolated cells for transfection, it remains a major challenge to apply it generally in a wide range of plant species due to difficulties in plant regeneration and obtaining a low frequency of genome-edited plants.

*Agrobacterium*-mediated transformation- and somatic protoplast-based genome editing has not been applicable to some plant species or cultivars; in contrast, the new system for directly delivering macromolecules to reproductive cells or tissues described here has the potential to be applied for producing genome-edited lines in a wide range of species or cultivars. Genome editing approaches by direct delivery of Cas9-gRNA RNPs into rice zygotes (*via* PEG-Ca^2+^-mediated transfection; Toda et al., 2019), cells of maize and wheat embryo cells (*via* particle bombardment; e.g., Svitashev et al., 2016; Liang et al., 2017), and cells of wheat SAMs (*via* particle bombardment; Kumagai et al., 2022) have been successfully established. Because foreign DNA sequences cause legislative concerns about genetically modified organisms (Jones 2015), the production of genome-edited rice, wheat, and maize *via* Cas9-gRNA RNPs is highly desirable for gene functional studies as well as for application to molecular plant breeding (Gu et al., 2021) and can reduce the frequency of off-target changes (Woo et al., 2015; Svitashev et al., 2016; Liang et al., 2017).

DSBs are mainly repaired *via* NHEJ pathways in the present methods, except for HDR-mediated gene editing in maize embryo cells (Svitashev et al., 2015). Thus, gene targeting *via* the HDR pathway can in principle be applied and function when Cas9-gRNA RNPs and donor DNA are delivered in reproductive cells/tissues. Although further optimization of procedures for preparation and delivery of RNPs-donor DNA components is required toward production of genome-edited lines possessing the donor DNA at the targeted genome site, these approaches using plant reproductive cells/tissues in various plant species or cultivars have the potential to accelerate a range of different research. This includes basic research, for example, functional analysis of genes of interest involved in reproductive and developmental events, such as gamete differentiation, fertilization, embryogenesis, and endosperm development in flowering plants, as well as applied sciences toward molecular plant breeding.
